# Combination treatment with transarterial chemoembolization, radiotherapy, and hyperthermia (CERT) for hepatocellular carcinoma with portal vein tumor thrombosis: Final results of a prospective phase II trial

**DOI:** 10.18632/oncotarget.17072

**Published:** 2017-04-13

**Authors:** Jeong Il Yu, Hee Chul Park, Sang Hoon Jung, Changhoon Choi, Sung Wook Shin, Sung Ki Cho, Dong Hyun Sinn, Yong-Han Paik, Geum-Youn Gwak, Moon Seok Choi, Joon Hyeok Lee, Kwang Cheol Koh, Byung Chul Yoo, Hüseyin Sahinbas, Seung Woon Paik

**Affiliations:** ^1^ Department of Radiation Oncology, Samsung Medical Center, Sungkyunkwan University School of Medicine, Seoul, Korea; ^2^ Department of Radiology, Samsung Medical Center, Sungkyunkwan University School of Medicine, Seoul, Korea; ^3^ Department of Medicine, Samsung Medical Center, Sungkyunkwan University School of Medicine, Seoul, Korea; ^4^ Department of Medicine, Konkuk University Medical Center, Konkuk University, Seoul, Korea; ^5^ Institute for Hyperthermia Research, Partner of the Marien Hospital Herne, Hospital of the Ruhr-University Bochum, Bochum, Germany; ^6^ Department of Medical Device Management and Research, SAIHST, Sungkyunkwan University, Seoul, Korea

**Keywords:** hepatocellular carcinoma, radiotherapy, hyperthermia, response, complication

## Abstract

**Background & Aims:**

This study was designed to evaluate the efficacy and safety of combination transarterial chemoembolization (TACE) followed by radiotherapy (RT) and hyperthermia (CERT) in hepatocellular carcinoma (HCC) with portal vein tumor thrombosis (PVTT).

**Methods:**

This single-institution, single-arm, prospective phase II study was performed from October 2013 to February 2016. The objective response rate (ORR) was evaluated at 3 months after CERT completion, and overall ORR was the primary end point.

**Results:**

During the study period, 69 of 77 patients who consented to participate underwent at least one session of hyperthermia and RT. More than half of the patients (39, 56.5%) complained of severe hyperthermia-related pain. The overall ORR was 43.5% (30/69), and the ORR of the RT target area was 69.6% (48/69). Liver function status was not significantly affected by CERT. Overall survival, local progression-free survival, and progression-free survival of all enrolled patients at 2 years was 62.9%, 47.6%, and 14.3%, respectively.

**Conclusions:**

An overall ORR of 43.5% was observed after CERT, but a promising ORR of 69.6% was achieved in the RT target area. Toxicities related to CERT were manageable, and pain intolerance to hyperthermia was the main obstacle to treatment maintenance.

## INTRODUCTION

Portal vein tumor thrombosis (PVTT), which is one of the most recognized prognostic factors of hepatocellular carcinoma (HCC), can lead to liver function deterioration and intrahepatic and/or distant metastasis, so the immediate elimination of PVTT is a key target for improving clinical outcomes [1–4]. Recently, the use of radiotherapy (RT) in HCC is increasing worldwide with rapid advancements in radiation physics and radiobiology [2, 5–7]. According to a recent meta-analysis, the 5-year survival rate was increased (hazard ratio [HR] of 3.92) with the addition of RT compared with transarterial chemoembolization (TACE) alone in the treatment of HCC with PVTT [8]. On the basis of these results, several guidelines recommend addition of RT for HCC with PVTT, including the Korean Liver Cancer Study Group, Singapore, and National Comprehensive Cancer Network [9–11].

Although there is clear evidence that greater local control can be obtained with RT dose escalation [12, 13], RT dose is largely limited by the baseline liver function, background cirrhotic liver status, extent of HCC, and/or proximity of the bowel. Therefore, a combination strategy that maximizes tumor control with a moderate RT dose is generally applied, especially with TACE [4, 8]. Our research group also reported favorable outcomes with acceptable toxicities for TACE plus RT in patients with HCC with PVTT [14]. However, the approximately 50% response rate of TACE plus RT due to RT dose limitation was not satisfactory.

Hyperthermia, which has direct cell-killing effects at temperatures above 41-42°C, is a well-known radiotherapy sensitizer [15]. Hyperthermia enhances the effects of RT in cancer cells, especially cells in S phase or under hypoxic and/or acidic conditions, which are generally considered to indicate radioresistant status. Hyperthermia can increase blood flow and enhance re-oxygenation of hypoxic tumors, which is a key challenge in the enhancement of RT effects [16]. Based on this theoretical background, hyperthermia has been applied in combination with RT on a wide range of lesions [17]. Several promising oncologic outcomes were reported with minimal adverse effects and adjacent tissue damage [18–21]. Furthermore, there have been several randomized controlled trials indicating that hyperthermia has a radiosensitizing effect in several cancer lesion types [22].

Based on the synergistic effects of RT and hyperthermia, we performed a prospective phase II trial evaluating the effectiveness and safety of TACE followed by RT and hyperthermia (CERT) in patients with HCC and PVTT. We previously reported initial outcomes focusing on the safety of this treatment [23].

In the present study, we sought evaluate the efficacy of CERT and confirm the safety of its application in the treatment of patients with HCC and PVTT.

## RESULTS

### Patients

**Figure 1:**
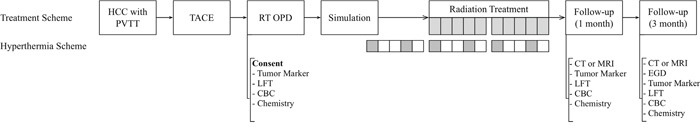
The schema of the study HCC, hepatocellular carcinoma; PVTT, portal vein tumor thrombosis; TACE, trans-arterial chemoembolization; RT, radiotherapy; OPD, outpatient department; QoL, quality of life; LFT, liver function test; CBC, complete blood count; CT, computed tomography; MRI, magnetic resonance imaging.

**Figure 2:**
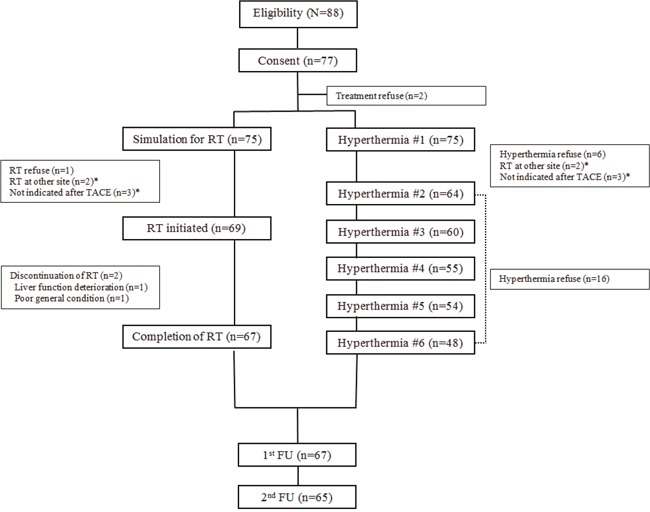
CONSORT diagram of the CERT study RT, radiotherapy; TACE, transarterial chemoembolization; FU, follow-up.

The schema of the present study is displayed in Figure 1. During the study period (October 2013 to February 2016), 77 of 88 eligible patients consented to participate in the present study. Among them, 75 patients underwent at least one session of hyperthermia, and 69 patients were treated with RT, 67 of whom completed RT. The CONSORT diagram is displayed in Figure 2.

### Treatment compliance of CERT

The characteristics of 69 patients who underwent at least one session of hyperthermia and RT are displayed in Table 1. Six patients (8.7%) were Child-Pugh class B, and 23 (33.3%), 42 (60.9%), and 4 (5.8%) were ALBI grades 1, 2, and 3, respectively. More than 70% of patients received 10 fractions of 3.0 or 3.5 Gy, and one patient failed to complete planned RT after five fractions due to persistent elevation of bilirubin after TACE. The calculated biologically effective dose (BED) ranged from 47.25 to 65.3 Gy (median 47.25 Gy, α/β = 10) in all except two patients (23.6 Gy in one patient who failed to complete RT after five fractions; 37.5 Gy in one patient who received off-protocol RT as 12 fractions of 2.5 Gy).

**Table 1:** Baseline characteristics of the study patients

Variables		Number of patients (%)
Age, years	Median Range	56 35 to 79
Sex	Male Female	60 (87.0) 9 (13.0)
ECOG performance status	0 1 2	36 (52.2) 29 (42.0) 4 (5.8)
Cause of hepatitis	HBV HCV Alcohol Other	57 (82.6) 6 (8.7) 4 (5.8) 2 (2.9)
Liver cirrhosis	Yes No	60 (87.0) 9 (13.0)
HCC diagnosis	Pathologic Clinical	10 (14.5) 59 (85.5)
Child-Pugh class	A B	63 (91.3) 6 (8.7)
ALBI grade	I II III	23 (33.3) 42 (60.9) 4 (5.8)
MELD score	Median Range	4 -1 to 12
No. of viable tumors	Solitary Multiple	32 (46.4) 37 (53.6)
mUICC T stage	T3 T4	34 (49.3) 35 (50.7)
mUICC N stage	N0 N1	67 (97.1) 2 (2.9)
mUICC M stage	M0 M1	67 (97.1) 2 (2.9)
Tumor size (cm)	Median Range	7.2 1.7 to 15.6
α-fetoprotein (ng/ml)	Median Range	163.0 1.6 to 200,000
Location of PVTT	Main First-order branch Second-order branch	15 (21.7) 32 (46.3) 22 (31.9)
Fraction size	2.5 Gy 3.0 Gy 3.5 Gy 4.0 Gy 4.5 Gy	1 (1.4) 25 (36.2) 24 (36.2) 15 (34.8) 4 (5.8)
Previous treatment (repeated measure)	Surgery RFA TACE Sorafenib Naïve	4 (5.9) 7 (10.1) 19 (27.5) 1 (1.4) 42 (60.9)

Abbreviations: ECOG, Eastern Cooperative Oncology Group; HBV, hepatitis B virus; HCV, hepatitis C virus; HCC, hepatocellular carcinoma; mUICC, modified International Union Against Cancer; PVTT, Portal vein tumor thrombosis; Gy, gray; RFA, radiofrequency ablation; TACE, transarterial chemoembolization.

### Treatment response

The primary endpoint of the present study was treatment response as assessed by mRECIST three months after completion of CERT. Among 69 patients, CR was achieved in 16 patients (23.2%), PR in 14 patients (20.3%), and PD in 33 patients (47.8%) at the time of response evaluation. Overall ORR, the primary endpoint of the present study, was 43.5%. However, when we focused on the RT target area, an objective response was achieved in 48 patients (69.6%): CR in 23 (34.0%) and PR in 25 (36.2%). Progression of disease in the RT target area was detected in 10 (14.5%) patients three months after CERT.

Typical cases showing a positive ORR after CERT are presented in Supplementary Figure 1.

### Probable prognostic factors of CERT response

Correlations between potential prognostic factors and overall or in-field ORR are displayed in Table 2. In terms of overall ORR, there were no statistically significant prognostic factors in the present study. Overall ORR was lowest in patients who were ALBI grade 3 (1/4, 25%), and maximum hyperthermia tolerance, accumulative hyperthermia energy, and total RT dose were not correlated with higher overall ORR. In contrast, there was a tendency toward higher RT in-field ORR with higher maximum hyperthermia tolerance (80.6% for ≥150 W), accumulative hyperthermia energy (82.1% for ≥200 J), and total RT dose (88.9% for BED ≥55 Gy), although a statistical significance was not reached.

**Table 2:** Potential factors related to overall objective response among 65 patients evaluated at Three months after completion of CERT

Variables		N	Overall		P	In-field		P
			Yes	No		Yes	No	
Gender	Male Female	56 9	23(41.1) 7 (77.8)	33 (58.9) 2 (22.2)	0.07	41 (73.2) 7 (77.8)	15 (26.8) 2 (22.2)	1.00
Age (years)	<55 ≥55	29 36	85 (47.2) 81 (54.5)	95 (52.8) 68 (45.6)	0.62	22 (75.9) 26 (72.2)	7 (24.1) 10 (27.8)	0.78
ECOG performance status	0-1 2	62 3	29 (46.8) 1 (33.3)	33 (53.2) 2 (66.7)	1.00	47 (75.8) 1 (33.3)	15 (24.2) 2 (66.7)	0.17
Liver cirrhosis	Yes No	57 8	25 (43.9) 5 (62.5)	32 (56.1) 3 (37.5)	0.46	41 (71.9) 7 (87.5)	16 (28.1) 1 (12.5)	0.67
Child-Pugh class	A B	59 6	28 (47.5) 2 (33.3)	31 (52.5) 4 (66.7)	0.68	44 (74.6) 4 (66.7)	15 (25.4) 2 (33.3)	0.65
ALBI grade	I II III	21 40 4	11 (52.4) 18 (45.0) 1 (25.0)	10 (47.6) 22 (55.0) 3 (75.0)	0.61	17 (81.0) 29 (72.5) 2 (50.0)	4 (19.0) 11 (27.5) 2 (50.0)	0.38
Tumor size	<5 cm ≥5 cm	15 50	7 (46.7) 23 (46.0)	8 (53.3) 27 (54.0)	1.00	13 (86.7) 35 (70.0)	2 (13.3) 15 (30.0)	0.32
Viable tumor	Single Multiple	31 34	13 (41.9) 17 (50.0)	18 (58.1) 17 (50.0)	0.62	23 (74.2) 25 (73.5)	8 (25.8) 9 (26.5)	1.00
Level of PVTT	Main Other	14 51	7 (50.0) 23 (45.1)	7 (50.0) 28 (54.9)	0.77	8 (57.1) 40 (78.4)	6 (42.9) 11 (21.6)	0.17
Pretreatment AFP level (ng/ml)	<200 ≥ 200	34 31	16 (47.1) 14 (45.1)	18 (52.9) 17 (54.8)	1.00	24 (70.6) 24 (77.4)	10 (29.4) 7 (22.6)	0.58
Total RT dose(BED with α/β=10)	<55 Gy ≥55 Gy	47 18	22 (46.8) 8 (44.4)	25 (53.2) 10 (55.6)	1.00	32 (68.1) 16 (88.9)	15 (31.9) 2 (11.1)	0.12
Maximum hyperthermia tolerance	<150 W ≥150 W	29 36	13 (44.8) 17 (47.2)	16 (55.2) 19 (52.8)	1.00	19 (65.5) 29 (80.6)	10 (34.5) 7 (19.4)	0.26
Accumulative hyperthermia energy	<2,000 J ≥2,000 J	37 28	17 (45.9) 13 (46.4)	20 (54.1) 15 (53.6)	1.00	25 (67.6) 23 (82.1)	12 (32.4) 5 (17.9)	0.26

Abbreviations: ECOG, Eastern Cooperative Oncology Group; PVTT, Portal vein tumor thrombosis; AFP, α- fetoprotein; RT, radiotherapy; BED, biologically equivalent dose; Gy, gray.

### Patterns of the first site of failure

During follow-up (median, 11.4 months; range, 2.1 to 30.5 months), 54 (78.3%) patients experienced recurrence. Among them, the first site of progression was local progression in 13 patients (18.8%) and intrahepatic progression in 32 patients (46.3%). Extrahepatic progression developed in 14 patients (20.3%) as the first site of recurrence. Among these patients, three had simultaneous local and intrahepatic recurrence, and two had simultaneous intrahepatic and extrahepatic recurrence. The pattern of first site of failure is displayed in Supplementary Figure 2.

### Survival outcomes

**Figure 3:**
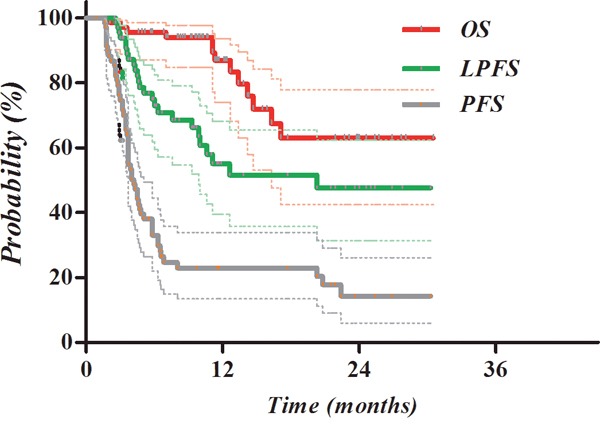
Kaplan-Meier survival curves after CERT Local progression-free survival LPFS), progression-free survival (PFS), and overall survival (OS) of all enrolled patients at 2 years were 62.9%, 47.6%, and 14.3%, respectively.

The Kaplan-Meier survival curves after CERT are displayed in Figure 3. OS, LPFS, and PFS of all enrolled patients at 2 years was 62.9%, 47.6%, and 14.3%, respectively.

**Figure 4:**
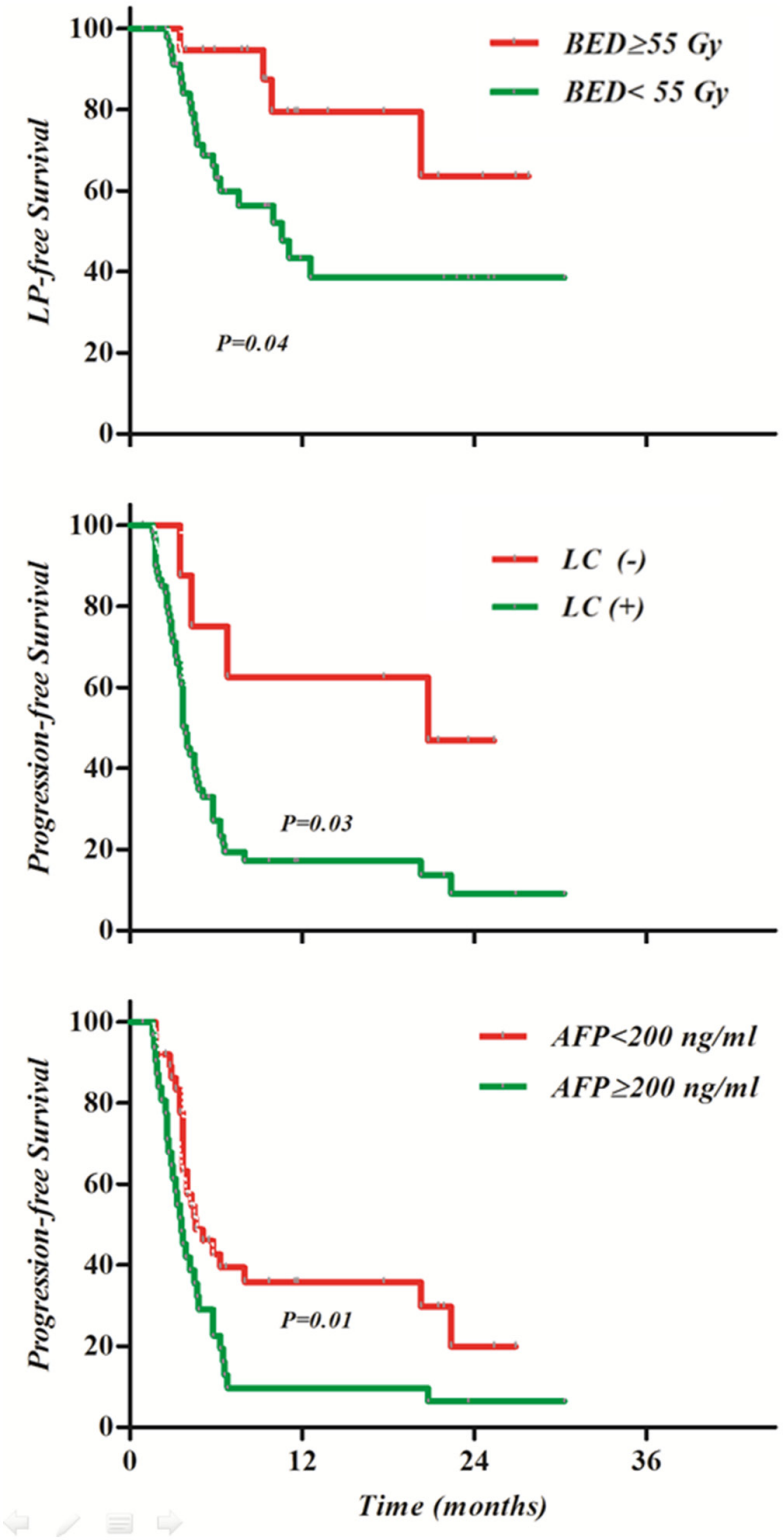
Kaplan-Meier survival curves according to prognostic factor **(A)** Biologically effective dose (BED) ≥55 Gy was a significant factor in local progression-free survival (LPFS); liver cirrhosis **(B)** and alpha fetoprotein (AFP) ≥200 ng/ml **(C)** were significant prognostic factors in progression-free survival (PFS).

The results of univariate analysis of probable prognostic factors of survival are shown in Table 3. On multivariate analysis, total RT dose of BED 55 Gy or greater was significantly associated with LPFS (P=0.04, HR 3.18, 95% confidence interval [CI] 1.07-9.49) and liver cirrhosis (P=0.03, HR 3.26, 95% CI 1.12-9.47), while pretreatment AFP >200 ng/ml (P=0.12, HR 2.12, 95% CI 1.20-3.75) was significantly associated with PFS (Table 4). Kaplan-Meier survival curves according to the above variables are shown in Figure 4. There were no significant prognostic factors of OS.

**Table 3:** Univariate analysis of probable prognostic factors of local progression-free survival LPFS), progression-free survival (PFS), and overall survival (OS)

Variables	LPFS			PFS			OS		
	HR	95% CI	*P*	HR	95% CI	*P*	HR	95% CI	*P*
Age (years) ≤ 55 *vs*. > 55	1.41	0.63-1.40	0.41	0.94	0.55-1.63	0.84	0.86	0.29-2.61	0.80
Sex Female *vs*. Male	5.38	0.72-40.11	0.10	1.09	0.51-2.34	0.82	3.12	0.40-24.26	0.78
ECOG performance status 0-1 *vs*. 2	2.53	0.33-19.24	0.37	2.12	0.65-6.90	0.21	2.53	0.32-20.14	0.38
Liver cirrhosis No *vs*. Yes	5.59	0.75-41.83	0.09	3.33	1.19-9.35	0.07	3.69	0.47-28.95	0.21
Child-Pugh class A *vs*. B	3.54	1.15-10.94	**0.03**	1.82	0.77-4.32	0.18	4.72	0.01-2063	0.62
ALBI grade 1 *vs*. 2	1.26	0.55-2.88	0.58	1.83	0.99-3.38	0.05	1.94	0.58-6.47	0.28
Tumor size (cm) <5 *vs*. ≥5	2.14	0.73-6.24	0.16	2.08	1.01-4.31	**0.05**	32.9	0.18-6015	0.19
Viable tumor single *vs*. multiple	1.25	0.57-2.75	0.57	1.40	0.81-2.42	0.23	3.33	0.92-12.12	0.07
Level of PVTT other *vs*. main	1.38	0.52-3.69	0.52	1.07	0.54-2.14	0.85	2.28	0.70-7.46	0.17
Pretreatment AFP (ng/ml) ≤ 200 *vs*. > 200	1.69	0.77-3.73	0.19	1.95	1.13-3.36	**0.02**	3.84	1.17-12.57	**0.03**
Total RT dose (BED with α/β=10) <55 Gy *vs*. ≥55 Gy	3.22	1.10-9.43	**0.03**	1.24	0.67-2.30	0.50	6.20	0.80-47.78	0.08
Maximum hyperthermia tolerance <150 W *vs*. ≥ 150 W	1.35	0.62-2.97	0.45	1.29	0.74-2.23	0.37	1.13	0.37-3.47	0.22
Total hyperthermia session <5 *vs*. ≥ 5	1.32	0.58-2.98	0.51	1.00	0.56-1.78	0.99	0.68	0.19-2.50	0.57
Accumulative hyperthermia energy <2,000 J *vs*. ≥2,000 J	1.15	0.251-2.55	0.74	1.22	0.71-2.11	0.48	1.66	0.55-4.96	0.37

Abbreviations: ECOG, Eastern Cooperative Oncology Group; PVTT, Portal vein tumor thrombosis; AFP, α-fetoprotein; RT, radiotherapy; BED, biologically equivalent dose; Gy, gray.

**Table 4:** Multivariate analysis of probable prognostic factors of local progression-free survival (LPFS), progression-free survival (PFS), and overall survival (OS)

Variables	LPFS			PFS			OS		
	HR	95% CI	*P*	HR	95% CI	*P*	HR	95% CI	*P*
Liver cirrhosis No *vs*. Yes	5.92	0.78-44.9	0.09	3.26	1.12-9.47	**0.03**			
Child-Pugh class A *vs*. B	2.29	0.73-7.18	0.16						
ALBI grade 1 *vs*. 2				1.51	0.80-2.86	0.20			
Tumor size (cm) <5 *vs*. ≥5				1.74	0.82-3.73	0.15			
Viable tumor single *vs*. multiple							1.75	0.45-6.86	0.42
Pretreatment AFP (ng/ml) ≤ 200 *vs*. > 200				2.12	1.20-3.75	**0.01**	2.51	0.72-8.68	0.15
Total RT dose (BED with α/β=10) <55 Gy *vs*. ≥55 Gy	3.18	1.07-9.49	**0.04**				3.93	0.49-31.69	0.20

Abbreviations: AFP, α-fetoprotein; RT, radiotherapy; BED, biologically equivalent dose; Gy, gray.

The ORR of CERT was a significant prognostic factor for PFS, for both RT target area (P=0.003) and overall (P<0.001). With regard to OS, however, only overall ORR was a significant prognostic factor (P=0.01).

### Adverse events

**Figure 5:**
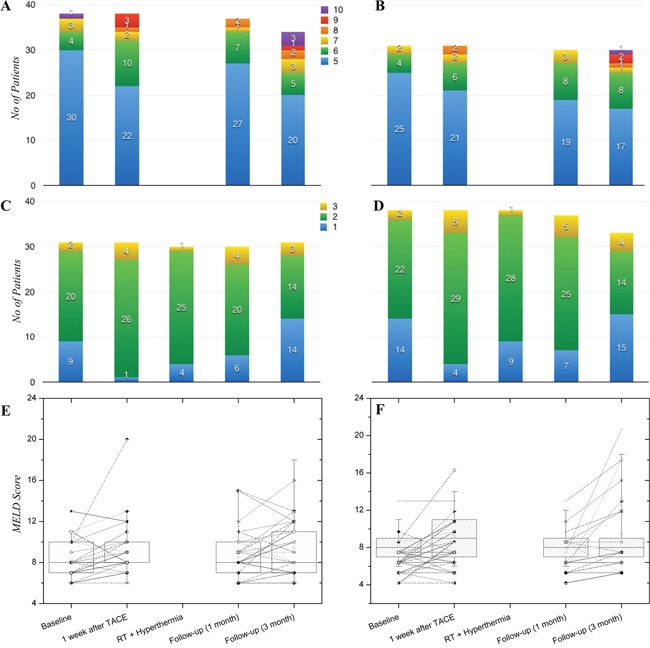
Changes in liver function status assessed by Child-Pugh score **(A, B)**, ALBI grade **(C, D)**, and MELD score **(E, F)**. Liver function status was slightly decreased after TACE, but recovered during RT and hyperthermia and was maintained at the one- and three-month follow-up visits after completion of CERT.

Liver function status assessed by Child-Pugh score, ALBI grade, and MELD score at baseline, one week after TACE, during RT and hyperthermia, and at the one-month and three-month follow-up visits after completion of CERT is displayed in Figure 5. Liver function status was slightly decreased one week after TACE, especially ALBI grade, but recovered during RT and hyperthermia. Severe deterioration of liver function as assessed by these indicators was not detected within one month after completion of CERT. This was also the case at the three-month follow-up visit, with the exception of elevated Child-Pugh score in patients who showed disease progression (P=0.009).

One patient did not finish planned RT and hyperthermia because of continuous elevation of total bilirubin after TACE. One patient died one month after CERT completion due to severe pneumonitis in the left lung of unknown origin that was probably unrelated to RT or hyperthermia.

As shown in Table 5, other acute toxicities were mainly confined to grade I or II, and the greatest obstacle to CERT was pain during hyperthermia. The timing of the first occurrence of hyperthermia-related severe pain that interrupted maintenance of treatment and the pain score of NRS are displayed in Supplementary Table 1. More than half of the patients (39, 56.5%) enrolled in the present study complained of severe hyperthermia-related pain around the energy range of 80 to 120 W. Only 11 patients (15.9%) reported no pain or an NRS pain score less than five during hyperthermia. A total of 21 patients (30.4%) refused further hyperthermia sessions, mainly because of hyperthermia-related pain, although every effort was made to complete treatment, including administration of short-acting opioids. Additionally, planned escalation of energy to 200 W failed in 45 patients (65.1%), and 23 patients (33.3%) received hyperthermia of only 100 W or less because of the pain.

**Table 5:** Acute toxicity profile during and after CERT

Grade	I (%)	II (%)	III (%)	IV (%)	V (%)
During CERT					
Fatigue	4 (8.7)	1 (2.2)	-	-	-
Anorexia	8 (11.6)	6 (8.7)	-	-	-
Nausea	9 (13.0)	2 (2.9)	1 (1.4)	-	-
Vomiting	2 (4.3)	-	1 (1.4)	-	-
Diarrhea	-	-	-	-	-
Abdominal pain	18 (26.1)	16 (23.2)	10 (14.5)	-	-
Acute toxicity during 3 months follow-up after CERT					
Anemia	14 (20.3)	10 (14.5)	-	-	-
Neutropenia	4 (5.8)	14 (20.3)	5 (7.2)		
Thrombocytopenia	33 (47.8)	14 (20.3)	5 (7.2)	-	-
AST	33 (47.8)	7 (10.1)	1 (1.4)	-	-
ALT	31 (44.9)	2 (2.9)	-	-	-
ALP	43 (62.3)	5 (7.2)	1 (1.4)	-	-
Albumin	12 (17.4)	8 (11.6)	-	-	-
Bilirubin	8 (11.6)	9 (13.0)	2 (2.9)		
Anorexia	7 (10.1)	4 (8.7)	-	-	-
Nausea	3 (4.3)	-	1 (1.4)	-	-
Vomiting	1 (1.4)	-	1 (1.4)	-	-
Diarrhea	1 (1.4)	-	1 (1.4)	-	-
Abdominal pain	10 (14.5)	3 (4.3)	5 (7.2)	-	-

Abbreviations: RT, radiotherapy; AST, aspartate aminotransperase; ALT, alanine aminotransperase; ALP, Akaline phosphatase; GGT, gamma-glutamyl transferase.

Within three months after completion of CERT, two patients (2.9%) were found to have gastroduodenal ulcers with pain and/or bleeding that required medical intervention. Additionally, asymptomatic radiation-related gastroduodenal ulcers were detected in 11 patients (15.9%), gastroduodenitis in two patients (2.9%), and erosion in six patients (8.7%) on preplanned routine follow-up EGD.

## DISCUSSION

The present study evaluating the efficacy of CERT, combination treatment with TACE followed by RT and hyperthermia, in patients with HCC and PVTT failed to reach the primary endpoint of 75% for overall ORR. However, when we focused on the RT target area, the ORR of CERT was 69.6%. Gastroduodenal toxicities were not uncommon, with an incidence of 18.8% after CERT on routine EGD follow-up, but symptomatic toxicities were detected in fewer than 5% of patients. Additionally, liver function assessed by Child-Pugh score, MELD score, and ALBI grade was generally maintained in patients treated with CERT, without evidence of disease progression.

Although there have been outstanding advancements in RT techniques and radiobiology, obstacles that prohibit the application of RT act as barriers to achieving a high response rate using RT in HCC management. Borderline background liver status is the biggest hurdle to achieving RT response. Gastroduodenal toxicity is another critical issue that inhibits physicians from delivering high-dose RT. Because of concerns about radiation-induced liver or gastroduodenal toxicities, the response rate of RT is limited to 40-60% of treated HCC patients, especially in patients with PVTT located at the mid-point of the liver and in close proximity to the gastroduodenum [2]. However, achieving a response might block liver function deterioration and maintain gastroduodenal integrity, which is also affected by appropriate liver function [4].

Hyperthermia is one of the oldest and most commonly used radiation sensitizers [27]. In thermal radiosensitization, hyperthermia kills hypoxic and S-phase cells, which are resistant to radiation therapy. It also enhances radiation response by inhibiting DNA damage repair. There are many clinical and experimental data available on the radiosensitizing effect of hyperthermia in cancer cells [15]. Thus, the addition of hyperthermia to combined treatment with TACE and RT is believed to confer additional benefit to patients. Recent preclinical studies using magnetic nanoparticles in animal liver cancer models revealed that magnetic fluid hyperthermia reduces tumor size with increased necrosis and no liver toxicity [28], and it also dramatically increases apoptotic rate and necrotic rate when combined with radiation therapy [29].

A similar complementary effect could be seen in the tumors of patients treated with CERT, although there was no histological analysis. In the present study, we confirmed the radiosensitizing effect of hyperthermia for achieving an in-field ORR of approximately 70%. The relatively high response rate of CERT is encouraging considering that more than 70% of patients enrolled in the present study received 10 fractions of 3.0 Gy or 3.5 Gy, which is a relatively low dose for achieving an acceptable ORR using RT alone. Although hyperthermia was combined with TACE and RT, liver function was well maintained during treatment and up to one month after CERT completion. Furthermore, the symptomatic gastroduodenal toxicity rate of 2.9% in the present study was relatively low compared with a rate of 24.0% in our previous study of TACE followed by RT [14]. These results suggest that the combination of hyperthermia with TACE followed by RT is effective for achieving a response rate and maintaining liver function and is safe in terms of radiation-induced liver disease and gastroduodenal toxicity.

Although a promising response rate of CERT was observed in the RT target area, there was a disappointing 43.5% overall ORR, which was similar or slightly below that of other reports for TACE-RT. Furthermore, overall ORR was the only significant prognostic factor for OS, which suggests that reducing intrahepatic progression is the crucial element in the management of HCC with PVTT. There could be several possible origins for this unsatisfactory finding of intrahepatic RT out-field progression after CERT.

Firstly, hyperthermia could enhance intrahepatic tumor recurrence by increasing serum levels of tumor growth factors, like vascular endothelial growth factor (VEGF) and matrix metalloproteinase (MMP), among others [30]. In a recent prospective phase II study using sorafenib, which is an oral multikinase inhibitor, and deep locoregional electro-hyperthermia in advanced HCC, promising PFS was reported, although no CR was observed. Combination therapy with a systemic target agent like sorafenib could be a reasonable solution to overcoming higher intrahepatic out-field progression after CERT [31].

Secondly, suboptimal and/or inhomogenous delivery of hyperthermia focusing on the iso-center of the RT field could explain the low ORR. Pretreatment simulation and/or real-time thermometry using MRI could minimize the uncertainty related to these problems [32].

Lastly, the effects of hyperthermia on intrahepatic out-field RT might be insufficient to reach the threshold of tumor cell killing because of low compliance. This could also be related to the abovementioned two problems. In the present study, optimal delivery of appropriate thermal energy was challenging. More than 50% of patients experienced severe pain during hyperthermia, and more than 60% of these patients did not complete the planned hyperthermia sessions. This result is similar to the findings of a previously reported phase II trial of RT and hyperthermia for unresectable HCC by Kim et al. [33], which showed acceptable local pain in 51.2% of patients. The positioning of the somatosensory system in the upper abdomen (skin, periosteum of the rib, parietal peritoneum and liver capsule) might be the reason for this poor compliance with hyperthermia in HCC. Additionally, although statistical significance was not achieved, considering the higher response rate in patients who showed higher maximum hyperthermia tolerance (80.6% for ≥ 150 W) or accumulative hyperthermia energy (82.1% for ≥ 200 J), further efforts to increase tolerance to hyperthermia are warranted to maximize the effects of CERT. Also, more focused delivery of hyperthermia with real-time thermometry is needed to enhance compliance in HCC.

The present study had several inevitable limitations. The results of a single-arm and single-institution study can only be generalized with caution. Furthermore, the actual temperature and/or absorption rate of the tumor and/or surrounding normal tissues during hyperthermia, which could more concretely illustrate the hyperthermia effect, were not assessed. Finally, even though 69 patients were enrolled and received CERT with follow-up, the present study did not reach the target sample size of 87 patients.

Although the primary end point of overall ORR of 75% was not achieved, a promising ORR of 69.6% for the RT target area was observed in the present study, with a relatively low incidence of liver and gastroduodenal toxicity. However, intolerance of pain related to hyperthermia was challenging to manage. A large multi-center prospective study to confirm the synergistic effects and safety of hyperthermia with TACE and RT is necessary, and methods of increasing pain tolerance in relation to hyperthermia should be investigated.

## MATERIALS AND METHODS

The present study was approved by the Samsung Medical Center Institutional Review Board in accordance with the ethical principles of the Declaration of Helsinki and local guidelines and was registered on clinicaltrials.gov with an identification number of NCT02290977.

### Patients

Subjects of this study were patients with diagnosed HCC according to the American Association for the Study of Liver Diseases guidelines combined with PVTT that was either present at the first presentation or developed during follow-up after treatment. PVTT was confirmed on computed tomography (CT) or magnetic resonance imaging (MRI) with the characteristic enhancement pattern of a portal venous intraluminal lesion. Detailed inclusion or exclusion criteria of the present study were described in our previous article [23].

### Study design

Patients that underwent superselective TACE and were eligible for CERT were identified, and written informed consent was obtained from all participants. Approximately one week after TACE, patients sequentially underwent follow-up liver function, a one-hour education session on respiration control, four-dimensional CT for RT planning, and the first session of hyperthermia. Planning MRI was also performed on the same day. Approximately one week after RT planning, 10 fractions of three-dimensional conformal RT were delivered with respiration gating. A total of six sessions of hyperthermia were performed after RT delivery at a frequency of twice per week.

### Radiotherapy

First, laboratory tests were performed and assessed. If the results were compatible with eligibility, the RT simulation process was started as follows. After obtaining non-contrast CT images, CT scans were repeated 25-30 seconds (arterial phase images) and 50-60 seconds (portal phase) after intravenous injection of contrast media (Visapaque 270™; GE healthcare, Buckinghamshire, United Kingdom; 2 mL/kg to a maximum of 200 mL) at a rate of 5 mL/second under exhale breath-hold. Next, a 4D-CT image was acquired with a Real-time Position Management system (Varian Medical Systems, Palo Alto, CA) and binned to 10 phase bins.

Additionally, MRI including respiration-gated end exhale phase images was performed using a Philips 3.0-T Achieva MR system® (Philips Medical Systems, Best, Netherlands). T1-weighted non-contrast and arterial (15–20 seconds), portal (45-50 seconds), and delayed (120-125 seconds) images were obtained with injection of dynamic intravenous contrast medium (Gadovist®; Schering AG, Berlin, Germany; 0.1 ml/kg) at a rate of 2 ml/second. Finally, respiration-triggered T2-weighted single-shot fast spin-echo images at end exhale phase were also obtained with a 5.0-mm slice thickness. The end-exhale phase of the four-dimensional CT image was manually registered to gated MRI exhale phase images using internal landmarks, including tumor, vessels of hilar region, or lipiodolized mass, by the radiation dosimetrist, and each fusion was validated by two radiation oncologists and a physicist.

The gross tumor volume (GTV) was defined as the abnormal area noted on the exhale phase of CT and MRI. The clinical target volume (CTV) was defined as GTV plus a 7-mm margin area within the liver, and the planning target volume (PTV) was determined as CTV plus a 5-mm margin.

RT planning was conducted using the exhale CT images. The fractionation was fixed at 10, and daily fraction size, determined according to our institutional guideline on the percentage of the normal liver volume irradiated with ≥50% of the prescribed dose, ranged from 3.5-5.0 Gy in 0.5 Gy intervals. In cases where the stomach, duodenum, and/or small bowel were exposed to the full irradiation dose, a 3.0 Gy daily fraction size was used to avoid gastroduodenal toxicity. The patients were treated using a NovalisTx (Varian Medical System, Palo Alto, CA, USA) 2-3 weeks after TACE. Daily image guidance was performed using cone-beam CT or orthogonal kilovoltage images.

### Hyperthermia

Hyperthermia was administered immediately after RT using a Celsius TCS electrohyperthermia electromagnetic device (Celsius42+ GmbH Company, Cologne, Germany) to minimize the toxicity of combination treatment. Hyperthermia was administered twice a week from one week after TACE and immediately after irradiation during RT. Hyperthermia sessions were separated by more than 48 hours. Six 60-minute hyperthermia sessions were administered with an energy escalation protocol from 40 to 200 W. The RT isocenter was used as the center for hyperthermia. Vital signs of the patient were measured before and after hyperthermia, and skin temperature was monitored continuously using three glass fiber-optic sensors (Celsius TempSens, Celsius42+ GmbH Company, Cologne, Germany).

### Response and toxicity evaluation and follow-up

During hyperthermia and/or RT, weekly assessment of all enrolled patients was performed by a radiation oncologist. Routine follow-up with laboratory and imaging evaluation was performed at 1 and 3 months after CERT completion, every 3 months for the first 24 months, and then every 6 months up to 5 years or until death. To maximize local control, additional TACE was recommended at one month for all patients except those who achieved CR. Triphasic liver CT or MRI and blood work were performed at each follow-up visit, and esophagogastroduodenoscopy (EGD) was assessed 3 months after treatment. Overall and infield responses were assessed using CT or MRI scans 1 and 3 months after treatment completion using the modified Response Evaluation Criteria in Solid Tumors (mRECIST) [24]. Toxicity was evaluated using the National Cancer Institute Common Terminology Criteria for Adverse Events version 4.0 [25]. Pain status was assessed via Numeric Rating System (NRS) score [26]. Pattern of failure was assessed at the first site of recognized failure during follow-up, and recurrence detected at two or more sites within one month was categorized as simultaneous recurrence.

### Statistics

The primary endpoints of the present study were objective response rate (ORR), calculated as the combined number of patients with complete response (CR) or partial response (PR) with treatment-related toxicities evaluated at three months after treatment completion. To obtain 75% overall ORR for CERT compared with historical data of 60% for TACE followed by RT, a sample of 87 patients was required assuming 10% dropout, 95% confidence, and 80% power. Secondary endpoints were local progression-free survival (LPFS), progression-free survival (PFS), and overall survival (OS). Survival duration was assessed from the date of TACE treatment to the date that the event was monitored or last follow-up. LPFS, PFS, and OS were estimated with the Kaplan-Meier method.

Probable prognostic factors of overall ORR were evaluated using a Chi-square test or Fisher's exact test. Sequential changes in tumor markers and liver function status were assessed by Child-Pugh score, Model for End-Stage Liver Disease (MELD) score, and ALBI grade using repeated measures ANOVA. The Cox proportional hazard model was used to determine significant prognostic factors impacting survival outcomes in univariate analysis, and only statistically significant variables (P<0.1) in univariate analysis were included in multivariate analysis. All calculations were performed using SPSS 22.0 software for Windows (SPSS, Chicago, IL, USA), and *P*<0.05 was considered statistically significant.

## SUPPLEMENTARY FIGURES AND TABLE



## Abbreviations

RT: radiotherapy
HCC: hepatocellular carcinoma
PVTT: portal vein tumor thrombosis
HR: hazard ratio
TACE: transarterial chemoembolization
CERT: combination treatment of TACE and hyperthermia
CT: computed tomography
MRI: magnetic resonance imaging
GTV: gross tumor volume
CTV: clinical target volume
PTV: planning target volume
EGD: esophagogastroduodenoscopy
mRECIST: modified Response Evaluation Criteria in Solid Tumors
NRS: Numeric Rating System
ORR: objective response rate
CR: complete response
PR: partial response
LPFS: local progression-free survival
PFS: progression-free survival
OS: overall survival
MELD: Model for End-Stage Liver Disease
BED: biologically effective dose
CI: confidence interval
OS: overall survival
DMFS: distant metastasis-free survival
LRFS: local recurrence-free survival
HR: hazard ratio
CI: confidence interval.
