# Supplementary material for: Combination treatment with transarterial chemoembolization, radiotherapy, and hyperthermia (CERT) for hepatocellular carcinoma with portal vein tumor thrombosis: Final results of a prospective phase II trial
Source: Oncotarget. 2017 Apr 13;8(32):52651–64. doi: 10.18632/oncotarget.17072 (PMC5581058; doi:10.18632/oncotarget.17072)
Supplement: Supplementary file 1 [file oncotarget-08-52651-s001.pdf]

Combination treatment with transarterial chemoembolization, radiotherapy, and hyperthermia (CERT) for hepatocellular carcinoma with portal vein tumor thrombosis: Final results of a prospective phase II trial

SUPPLEMENTARY FIGURES AND TABLE

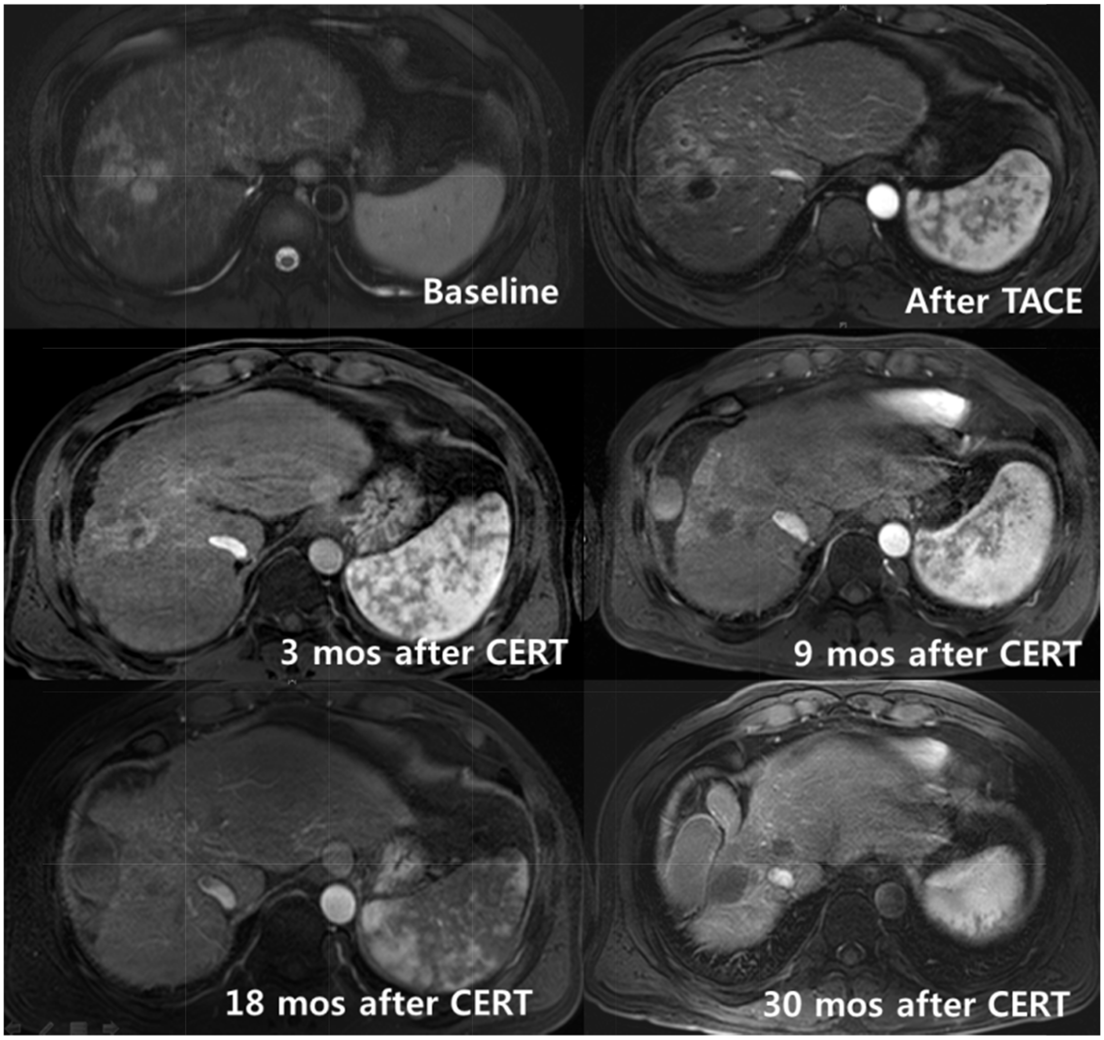

Supplementary Figure 1: The magnetic resonance (MR) imaging of typical case showing positive objective response after CERT. Forty-seven years old man was diagnosed with 4 cm hepatocellular carcinoma with tumor thrombosis on right anterior portal vein, and his initial AFP level was 15011.8 ng/ml. After TACE, he received RT (35 Gy/10 fraction) and hyperthermia (max 200W, accumulated energy 2248.1 J). He showed no viable tumor on MR image after planned additional TACE at 2 months after CERT and there was no evidence of recurrence on last follow up.

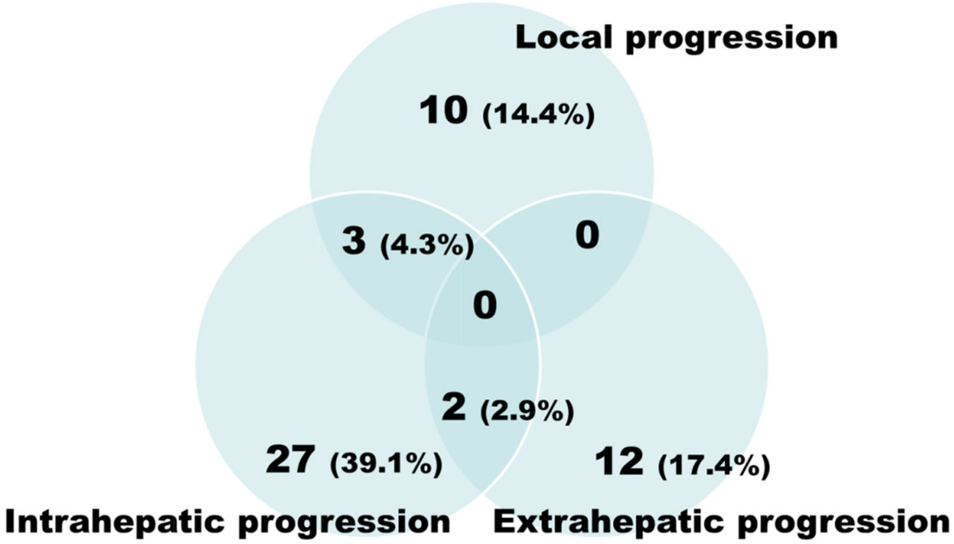

Supplementary Figure 2: Pattern of first site failure diagram. Intrahepatic recurrence was the most prominent progression pattern after CERT.

Supplementary Table 1: Timing and pain score of first attack of hyperthermia-related severe pain occurrence obstacle of treatment maintaining

NRS	5	6	7	8	9	10
40 W	-	-	-	-	-	-
60 W	-	-	-	2	1	1
80 W	-	-	1	4	3	1
100 W	-	3	4	5	6	5
120 W	-	-	1	3	2	1
140 W	-	-	1	-	-	-
160 W	-	1	2	3	3	2
180 W	-	-	-	-	-	-
200 W	-	-	-	-	3	1

NRS, numeric rating scale; W, watt.
